# Effects of a Novel Dexamethasone Hydrogel Drug Delivery System on Cytokine and Mucin Expression in a Three-Dimensional In Vitro Conjunctival Inflammation Model

**DOI:** 10.1167/iovs.67.1.42

**Published:** 2026-01-20

**Authors:** Julian Schwebler, Raoul Verma-Fuehring, Niloofar Kalantari, Constantin Berger, Daniel Kampik, Jost Hillenkamp, Malik Salman Haider, Christian Lotz

**Affiliations:** 1 Department for Functional Materials in Medicine and Dentistry, University Hospital Würzburg, Würzburg, Bavaria, Germany; 2 Translational Center Regenerative Therapies (TLC-RT), Fraunhofer Institute for Silicate Research (ISC), Würzburg, Bavaria, Germany; 3 Department of Ophthalmology, University Hospital Würzburg, Würzburg, Bavaria, Germany

**Keywords:** 3D conjunctiva model, inflammation, dexamethasone, hydrogel, POx

## Abstract

**Purpose:**

Inflammation of the ocular surface is one of the key symptoms in dry eye disease (DED). Various eye drops are available to alleviate conjunctival inflammation but have limited effect and poor patient compliance. To facilitate testing of new treatments, we established a three-dimensional (3D) in vitro conjunctival inflammation model and tested a novel dexamethasone (Dex) hydrogel drug-delivery system on cytokine and mucin expression.

**Methods:**

Primary human conjunctival fibroblasts were embedded in a compressed collagen matrix. Primary epithelial cells were seeded on top and cultured at the air-liquid-interface for 15 days. Inflammation was induced via TNF-α, IL-1β, or their combination. Pristine Dex, poly(2-oxazoline) (POx)–based Dex micelles and modified acrylic acid based hydrogel loaded with Dex micelles were applied to the inflammation model to verify the therapeutic efficacy. Pro-inflammatory cytokines were analyzed via real-time quantitative PCR (RT-qPCR) and ELISA. Conjunctival mucins were analyzed via RT-qPCR.

**Results:**

The expression of all tested cytokines (*IL1A, IL1B, IL6, IL8, MMP9*) was significantly increased after combined stimulation with TNF-α and IL-1β. Treatment with Dex-formulations significantly reduced *IL6* expression. The expression of mucins was significantly increased after stimulation and was further elevated after treatment with Dex-formulations.

**Conclusions:**

We established an inflammation model, in which the effects of novel treatment options on cytokine and mucin expression can be analyzed. This opens a new in vitro test platform for DED medication and enables deeper investigations into conjunctival mucin expression in inflamed tissue.

Dry eye disease (DED) is a global problem that affects at least 344 million people worldwide and is one of the main reasons for patients’ visits to the ophthalmologist.^1^^,^^2^ Independent of the DED subtype, one of its most common symptoms is inflammation of the ocular surface.^3^ These inflammatory responses can become chronic and cause discomfort, blurry vision, and, in severe cases, keratinization of the ocular surface.^4^ Another finding that has been reported in DED patients is a loss of goblet cells and alterations of mucin expression.^5^ The loss of conjunctival goblet cells results in a reduced expression of the gel-forming mucin MUC5AC^5^^–^^7^ along with an altered expression of membrane-associated mucins (MAMs).^8^^–^^10^ Sialylation of MUC1 has been reported to be increased in patients with mild to moderate DED and decreased in patients with severe DED as a possible compensatory reaction to the reduced MUC5AC.^5^^,^^11^ Although reduced MAMs have been reported in DED, other studies show that TNF-α can increase the expression of MUC1 and MUC4.^12^ Nevertheless, the exact interplay of inflammation and mucus expression mechanisms in conjunctival tissue remains poorly understood.

Although medications for inflammation such as glucocorticoids like dexamethasone (Dex) applied as eye drops, have been developed to dampen inflammatory responses, their therapeutic efficacy is limited because of low bioavailability and poor patient compliance.^13^^–^^15^ Additionally, the low solubility and permeability of Dex hinders its therapeutic application, which results in necessarily increased application frequencies.^16^^,^^17^ Furthermore, the effect of these drugs is often solely measured by their anti-inflammatory effect. Given the evidence for alterations of mucus in DED patients, regenerative effects on mucin expression should also be taken into consideration when evaluating DED treatments.

The limited bioavailability of glucocorticoids calls for alternative methods of administration. One interesting approach to improve the solubility and permeability of drugs with a poor bioavailability are micellar formulations. Polymeric micelles are nanoscopic self-assembled aggregates that have been investigated as drug delivery systems because of their advantages like nanosize and enhanced adhesion to cells.^18^ Hydrogels are of increasing interest for drug delivery because of their slow release properties.^19^^–^^21^ For ophthalmic use, the application of hydrogels that enable a slow release of drugs promises to improve patient compliance by lowering application frequencies.^22^ At the same time, the improved bioavailability of drugs loaded in hydrogels compared to standard eye drops may increase drug efficiency.^23^ To test these new treatment options on in vitro models, the models should resemble physiological properties as closely as possible while expressing the specific markers the medication aims to regulate. Previously, we have established a conjunctival model that shows physiological resemblance, including a connective tissue equivalent, as well as a non-keratinized stratified epithelium with goblet cell differentiation.^24^ To our knowledge, there is currently no conjunctival inflammation model that comprises these factors and in which the expression of DED inflammatory markers, conjunctival MAMs and secreted mucins were analyzed.

In this study, we aimed to develop a novel three-dimensional (3D) in vitro conjunctival inflammation model as a test platform for ophthalmic drug development. Additionally, we compared the effects of three different Dex treatments on the model: Pristine Dex, a Dex-loaded micellar formulation, and a slow-releasing Dex-loaded modified hyaluronic hydrogel.^25^ Previously, the Poly(2-oxazoline) (POx) based ABA triblock copolymers, which have hydrophilic and hydrophobic blocks, showed great potential for developing ultra-high drug-loaded micellar formulations.^26^^,^^27^ Specifically, ABA triblock copolymer with poly(2-methyl-2-oxazoline) (pMeOx) as the hydrophilic A block and moderately hydrophobic poly(2-n-butyl-2-oxazoline) (pBuOx) as the B block exhibit high drug loading, excellent cytocompatibility, and good tolerance in vivo.^28^ We have deployed this polymer to improve the solubility of highly hydrophobic Dex followed by its incorporation into the acrylate modified hyaluronic acid based hydrogels.^25^ In this study, we used this formulation as a treatment for our successfully established conjunctival inflammation model, which resulted in the alleviation of its inflammatory state. At the same time, the treatment increased the expression of MAMs in our model. This confirms POx-micelles–loaded hydrogels to be a suitable drug delivery system (DDS) for ocular surface disorders.

## Methods

### Human Tissue

All experiments conducted in this study followed the Declaration of Helsinki. Use of human material was approved by the local ethics committee (IRB of the Medical Faculty of the University of Wuerzburg; approval number 187/17, 195/13 and 182/10). Human conjunctival biopsies were obtained from the Department of Ophthalmology, University Hospital Würzburg, Germany, from adult patients either after their informed consent, or from deceased organ donors whose eyes were enucleated for corneal graft preparation. The tissue used for this study was derived from eyes with no further ocular pathology that affects the conjunctiva such as pterygium, pemphigoid, or significant inflammation. Biopsy specimens were anonymized and used within 24 hours for cell isolation. The human tissue experiments complied with the guidelines of the ARVO Best Practices for Using Human Eye Tissue in Research (Nov2021). For the experiments included in this study, epithelial cells and fibroblasts from five different donors were used.

### Isolation and Culture of Primary Conjunctival Cells

Primary human conjunctival cells were isolated, and full-thickness conjunctiva models (FTConM) were generated as previously described.^24^ On day 15 of culture, the models were stimulated by adding the respective stimulant into the medium of the basolateral compartment. To compare the effects of TNF-α and IL-1β, the models were stimulated for 24 hours with either 50 ng/mL TNF-α, 10 ng/mL IL-1β (both PeproTech Proteins; Thermo Fisher Scientific, Waltham, MA, USA), or their combination in the same concentrations. For treatment experiments with Dex-formulations, the models were initially stimulated with 50 ng/mL TNF-α + 10 ng/mL IL-1β. After four hours, the respective Dex treatment was added, and the models were cultured for another 20 hours. Treatments were applied to the basolateral compartment to not disturb differentiation processes caused by the air-liquid-interface culture. Cells were cultured at 37°C, 95% humidity, 5% CO_2_.

### Micelle and Hydrogel Formulation

The preparation of Dex-loaded micellar formulation has been described previously (Raoul Verma-Fuehring, IOVS, 2024, Vol. 65, ARVO E-Abstract, #1924). In short, a thin film of Dex and poly(2-oxazoline)s polymer (i.e., A-pBuOx-A via solvent evaporation) was prepared, followed by lyophilization, heat treatment at 240°C, and rapid quenching in liquid nitrogen for 60 seconds. The film was then rehydrated, vortexed, and spun in a centrifuge to remove precipitates, if any. All formulations were prepared in triplicate. The polymer to Dex ratio was kept at 10:2 g/L. UV-Vis spectroscopy and HPLC were used to quantify Dex.

For the slow-release hydrogel carrier, the micellar Dex-formulation was set into a hydrogel from an acrylate-based derivative of hyaluronic acid^25^ at a concentration of 0.08 g/L (200 µM) per disk, based on a 50% release observed in previous experiments over 24 hours. For inflammation experiments, the tested treatment groups included pristine Dex (100 µM), Dex-loaded POx polymer micelles (100 µM), Dex-loaded POx polymer micelles embedded in the hydrogel disk (200 µM), and blank POx polymer micelles (0 µM).

### HPLC

Chromatographic analyses were performed using a Nexera HPLC equipped with a system controller SCL-40, on-line degassing unit DGU-405, a solvent delivery pump LC-40D XR (parallel double plunger, 70 MPa pressure limit), an auto-sampler SIL-40C XR, CTO-40C column Oven (40°C), SPD-M40 photodiode array detector, wavelength range 190–800 nm. As stationary phase, Shim-pack GISS-HP C18; particle size 3 µm; length 150 mm × internal diameter 3.0 mm was used. The flow rate was kept constant at 1 mL/min whereas the injection volume was 10 µL. Quantification of Dex was performed with a stepwise gradient using acetonitrile and water with 0.05% TFA (mobile phase). Initially the HPLC method was developed for Dex. After that, the series of standard solutions of different known concentrations was measured to obtain the standard curve followed by the Dex quantification at its respective lambda max (248 nm). The retention time for the Dex was found to be 1.7 minutes. LabSolutions software version 5.124 was used for hardware control and data manipulation. Before the analysis of the samples, the system was equilibrated with the developed method using the blank sample.

### Optical Coherence Tomography

For noninvasive evaluation of FTConM cross-sections, optical coherence tomography (OCT) was used. OCT was performed with the Ganymede Series Spectral Domain OCT Imaging System (Thorlabs, Newton, NJ, USA). FTConM were analyzed in a two-dimensional scan with 6 mm sections across the center of the model.

### MTT-Viability Assay

Viability of the FTConMs after cytokine stimulation was assessed using an MTT-assay (3-[4,5-dimethylthiazol-2-yl]-2,5 diphenyl tetrazolium bromide). FTConM were incubated at 37°C in 2.5 mL MTT-solution (1 mg/mL) for three hours. After incubation, the models were transferred into a 12-well plate, and 3 mL of isopropanol was added. After 24 hours of incubation at 4°C, 200 µL was transferred into a 96-well plate in duplicates. The samples were analyzed using colorimetric measurement of absorbance at 570 nm (Tecan plate reader M Infinite nano; Tecan, Männedorf, Switzerland).

### Embedding and Histological Staining of FTConM

FTConM were fixed in Rotifix (Carl Roth, Germany) for three hours at RT. The models were washed in dH_2_O, following an ethanol/isopronanol series (50%, 70%, 80%, 90%, isopronanol I/II, Xylene I/II). The models were subsequently embedded in paraffin. Paraffin blocks were cut into 3.5 µm sections. Before staining, sections were incubated at 60°C to melt the paraffin from the tissue sections. The slides were transferred into a descending ethanol series (96%, 96%, 70%, 50%, dH_2_O) and stained with hematoxylin and eosin (H&E) following the manufacturer’s protocol (Morphisto, Offenbach am Main, Germany). Slides were mounted with Entellan (Merck, Darmstadt, Germany). For immunofluorescent staining of Ki67, serial sections with a thickness of 3.5 µm and a spacing of 20 µm were prepared. After blocking with 5% BSA, Ki67 primary antibody (ab16667, dilution 1:200; Abcam, Cambridge, UK) was applied overnight at 4°C. On the following day, the samples were washed three times with PBST (PBS^-^ + 0.5% Tween-20). The secondary antibody (AlexaFluor 555, dilution 1:400; LifeTechnologies, Carlsbad, CA, USA) was subsequently applied for one hour at room temperature. The slides were then mounted with FluoroMount (Fluoromount-G with DAPI; Thermo Fisher Scientific). For quantitative Ki67 analysis, whole model sections were captured and stitched on a Keyence BZ-X800 system (Keyence, Osaka, Japan). The stitched images were then analyzed on DAPI and Ki67 positive signals via IMARIS software (Oxford Instruments, Abingdon, UK).

### RT-qPCR

FTConM were lysed in TRK buffer (VWR International, Radnor, PA, USA) supplemented with 20 µL/mL β-Mercaptoethanol and homogenized in a Tissue lyser (Qiagen, Hilden, Germany). Total RNA was extracted using a peqGOLD Total RNA Kit (VWR International) according to the manufacturer's instructions. Concentration and purity of isolated RNA was measured via a NanoDrop One spectrophotometer (Thermo Fisher, Waltham, USA). Using the iScript cDNA Synthesis Kit (Bio-Rad, Hercules, California, USA), 1 µg of total RNA was reverse transcribed into cDNA. RT-qPCR was performed using the SsoAdvanced Universal SYBR Green Supermix (Bio-Rad Life Science, Hercules, CA, USA). All runs were performed with 60°C annealing temperature using the QuantStudio 7 Flex system (Thermo Fisher Scientific). Gene expression was calculated using the ΔΔCT-method with *RPL13A* as housekeeper reference. Primer sequences (Eurofins Scientific, Luxembourg City, Luxembourg) were used as stated in the Table.

**Table.** Genes and Primer Sequences Used for RT-qPCR

Gene	Gene Function	Primer Sequence (5′- 3′)
*RPL13A*	Housekeeping	F-GAAGCCTACAAGAAAGTTTGCC
		R-TGGTACTTCCAGCCAACCTC
*IL1A*	Inflammation	F-CAACCAGTGCTGAAGGAG
		R-TGCCGTGAGTTTCCCAGAAG
*IL1B*	Inflammation	F-CTGTCCTGCGTGTTGAAAGA
		R-TGGGTAATTTTTGGGATCTACA
*IL6*	Inflammation	F-GTCCACTGGGCACAGAACTT
		R-AAACTGCATAGCCACTTTCCA
*IL8*	Inflammation	F-GTCCTTGTTCCACTGTGCCT
		R-GCTTCCACATGTCCTCACAA
*MMP9*	Inflammation	F-TTCCAAACCTTTGAGGGCGA
		R-CAAAGGCGTCGTCAATCACC
*MUC1*	MAM	F-TGTCAGTGCCGCCGAAAGAA
		R-CTACAAGTTGGCAGAAGTGG
*MUC4*	MAM	F-GACTTGGAGCTCTTTGAGAATGG
		R-TGCAATGGCAGACCACAGTCC
*MUC5AC*	Gel-forming mucin	F-GGTGTTCTTTGACTGCCGAAATG
		R-GTCATGTCCAGTGTGTGGCAG
*MUC16*	MAM	F-GCCTCTACCTTAACGGTTACAATGAA
		R-GGTACCCCATGGCTGTTGTG
*TFF3*	Mucus stabilizing protein	F-CCCCTGCAGGAAGCAGAATG
		R-AACAGTGCCTGGCAGCAATC

### Statistical Analysis

For statistical analyses, GraphPad Prism 10 software (GraphPad Software Inc., La Jolla, CA, USA) was used. Data points for Ki67 staining, MTT-assays, RT-qPCR, and ELISA were analyzed for normality using the Shapiro-Wilk test. Statistical significance was analyzed via One-way ANOVA tests with Dunnett's test for multiple comparisons. For data sets that were not normally distributed a Kruskal-Wallis test was performed. Values of *P* < 0.05 were considered significant. Experiments were performed at least as *n* = 3. Dex-formulations were tested as *n* = 6 whereas blank micelles were tested as *n* = 5. Outliers in experiments were detected via 1.5 interquartile range (1.5 IQR).

## Results

### Stimulation With Pro-Inflammatory Cytokines Does Not Affect Model Viability

**Figure 1.**
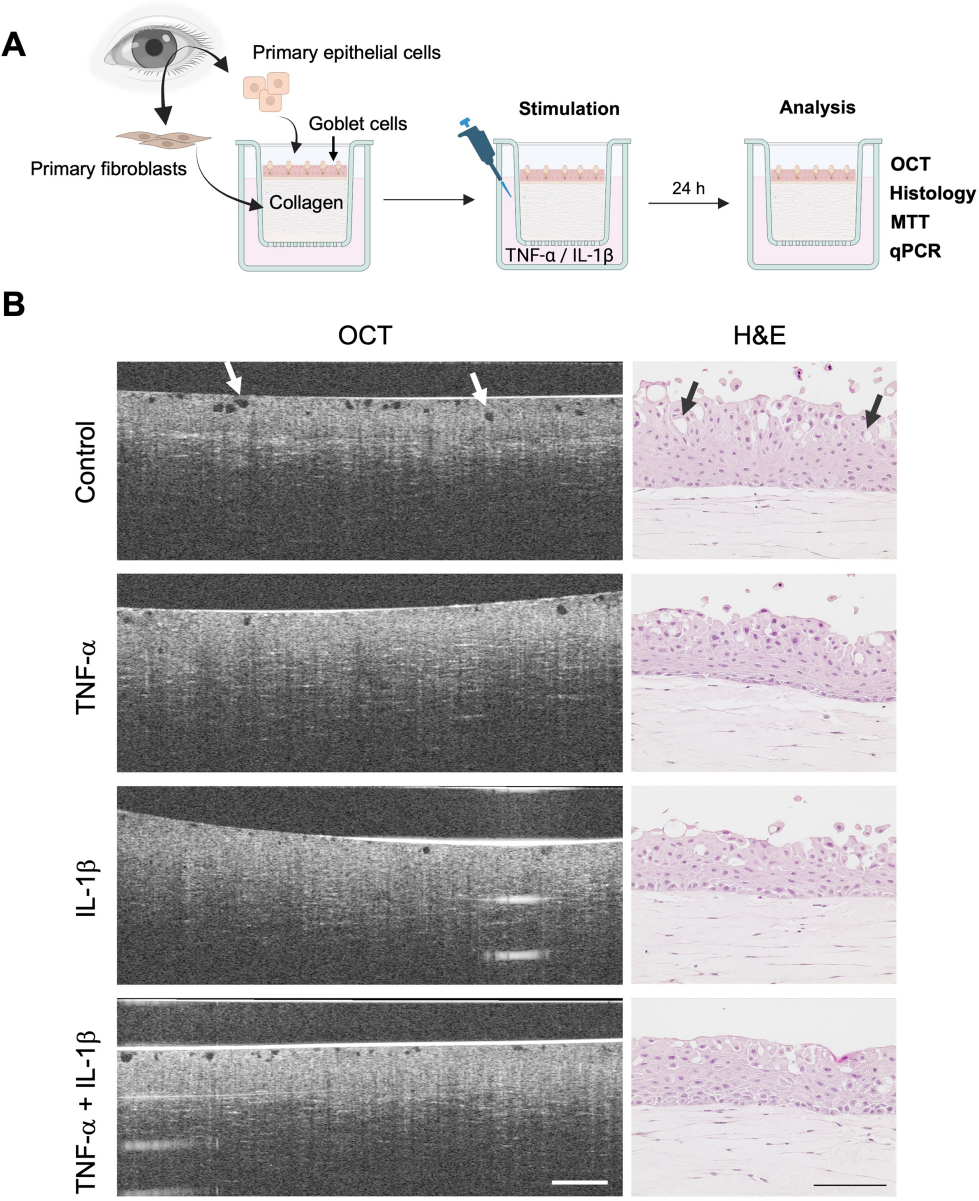
Stimulation with inflammatory cytokines does not affect model viability. **(A)** Schematic representation showing the setup of the FTConM and the treatment method. Created in BioRender. Salman Haider, M. (2026) https://BioRender.com/bexgla0 **(B)** Images of OCT and H&E staining of sections of an untreated control, after TNF-α, IL-1β, and TNF-α + IL-1β stimulation. *OCT scale bar*: 250 µM; *H&E scale bar*: 100 µM.

Cytokines are commonly used to induce inflammation in the in vitro conjunctiva models.^29^^,^^30^ TNF-α and IL-1β are known to be directly linked to the NF-κB pathway, which is one of the most important mediators in inflammatory responses. Although both cytokines activate NF-κB, it has been shown that TNF-α and IL-1β differentially influence NF-κB activity.^31^^,^^32^ As a first step within this study, we tested the effects of the cytokines (Fig. 1A). After 24 hours of stimulation, H&E staining revealed no observable disruption of the connective tissue equivalent nor the epithelial layers. OCT images were captured after inflammation and compared to H&E staining to establish a non-invasive imaging technique for FTConM analysis. OCT images revealed an intact epithelium, characterized by a brighter reflection compared to the connective tissue equivalent lying underneath (Fig. 1B). Dark round spots within the epithelium correlated with the location and distribution of putative goblet cells in histological sections. (Fig. 1B, *arrows*).

### Pro-Inflammatory Cytokines Reduce Ki67 in FTConM

**Figure 2.**
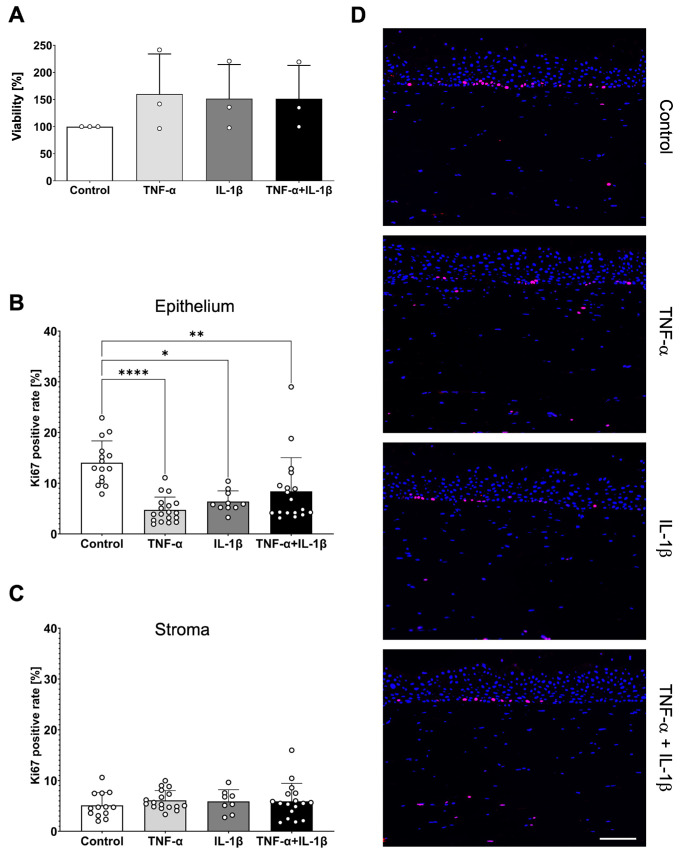
Inflammation decreases proliferation in the model. **(A)** MTT-assay of an untreated control versus cytokine stimulation. **(B)** Quantitative analysis of Ki67 staining in the model's epithelium. **(C)** Quantitative analysis of Ki67 in the model's stroma. **(D)** Immunofluorescence of models treated with TNF-α, IL-1β, or TNF-α + IL-1β. *Blue* = DAPI; *red* = Ki67. *Scale bar*: 100 µM. *P < 0.05, **P < 0.01, ***P < 0.001, ****P < 0.0001.

In line with the optical analyses, MTT assay confirmed the viability of FTConM after stimulation (Fig. 2A). The assay showed that viability was neither significantly affected by TNF-α (*P* = 0.48), nor IL-1β (*P* = 0.58) and their combination (*P* = 0.59) compared to the untreated control. For additional analyses on possible effects of the applied cytokines on hyperplasia, a Ki67 staining was subsequently performed (Figs. 2B–D). Interestingly, in cytokine treated models less positive signal within epithelial nuclei was detected. This was additionally approved via quantitative analyses of stained sections using IMARIS software. The model's inflammatory state significantly reduced the number of Ki67 positive epithelial cells (Fig. 2B) whereas the Ki67 rate in stromal cells remained unchanged (Fig. 2C).

### TNF-α and IL-1β Have Distinct Effects on Inflammation Markers

**Figure 3.**
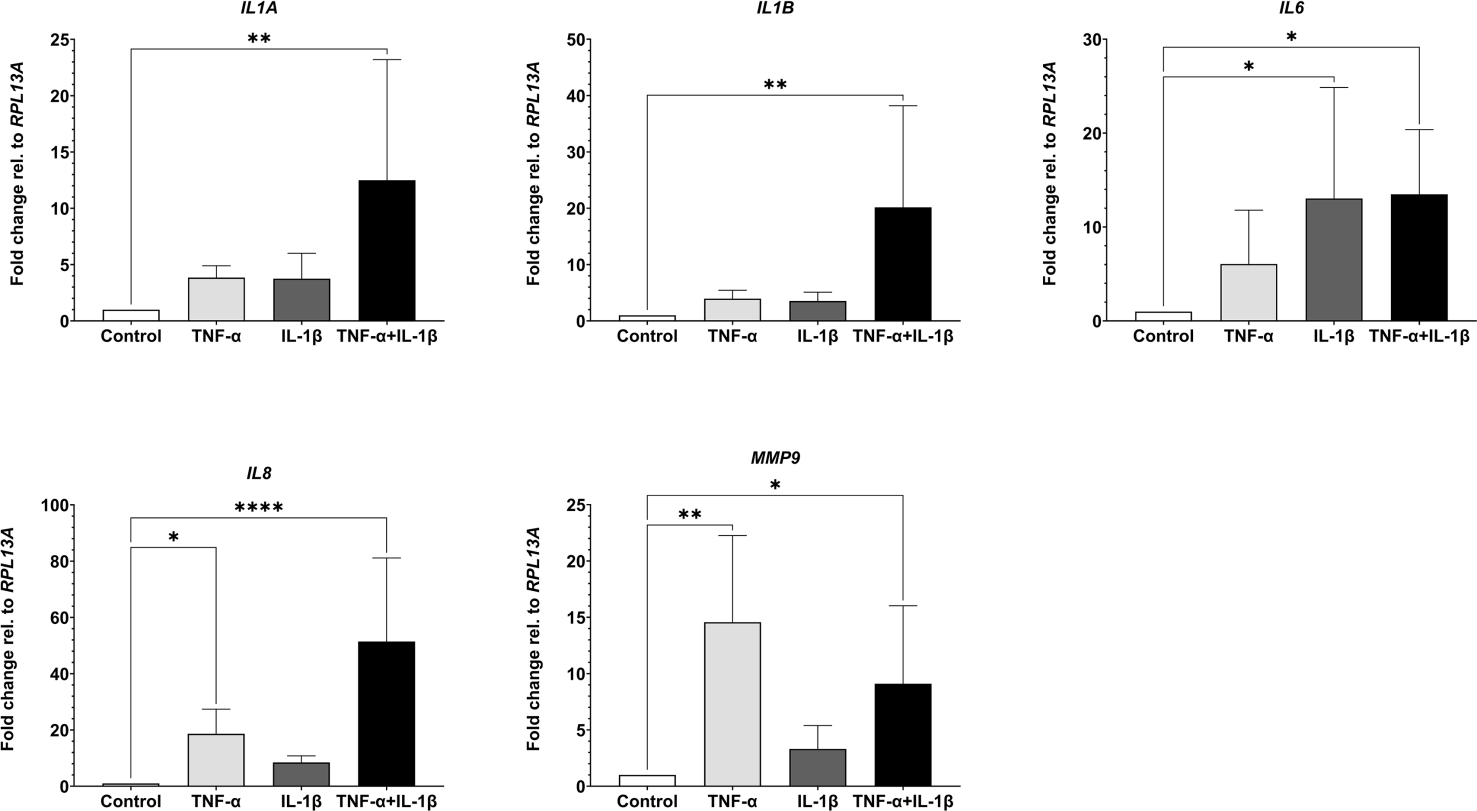
RT-qPCR of DED associated, pro-inflammatory markers after stimulation with TNF-α, IL-1β, or TNF-α + IL-1β. The application of TNF-α and, IL-1β had distinct effects on IL6, IL8, and MMP9, whereas their combination showed a synergic effect on the tested markers. *P < 0.05, **P < 0.01, ***P < 0.001, ****P < 0.0001.

To further assess the effect of the cytokines on the model, inflammatory markers known to be elevated in DED (*IL1A*, *IL1B*, *IL6*, *IL8*, *MMP9*) were analyzed via RT-qPCR (Fig. 3).^33^^–^^35^ The combinatory administration of TNF-α and IL-1β resulted in a significant increase of all DED-associated inflammatory markers, whereas the most dominant increase was observed for *IL8* expression. In contrast, stimulation with either TNF-α or IL-1β alone led to a less pronounced cytokine expression. Stimulation with TNF-α significantly increased *IL8* and *MMP9* expression, but had no significant effect on *IL1A*, *IL1B* and *IL6*. On the opposite, IL-1β administration increased *IL6*, but not *IL1A*, *IL1B*, *IL8* or *MMP9* expression. Comparing the expression of combinatorial and separate administration of TNF-α or IL-1β, we identified TNF-α as the main driver of *MMP9* expression and IL-1β as the most potent factor for the induction of *IL6* expression. The increased expression of *IL1A*, *IL1B* and *IL8* was a result of the synergistic effects of TNF-α and IL-1β. Summarized, the data demonstrate that the combinatory stimulation of TNF-α and IL-1β can be used to induce a DED-like inflammatory condition in FTConM.

### Full-Thickness Conjunctiva Model as a Test Platform for New Treatment Options

**Figure 4.**
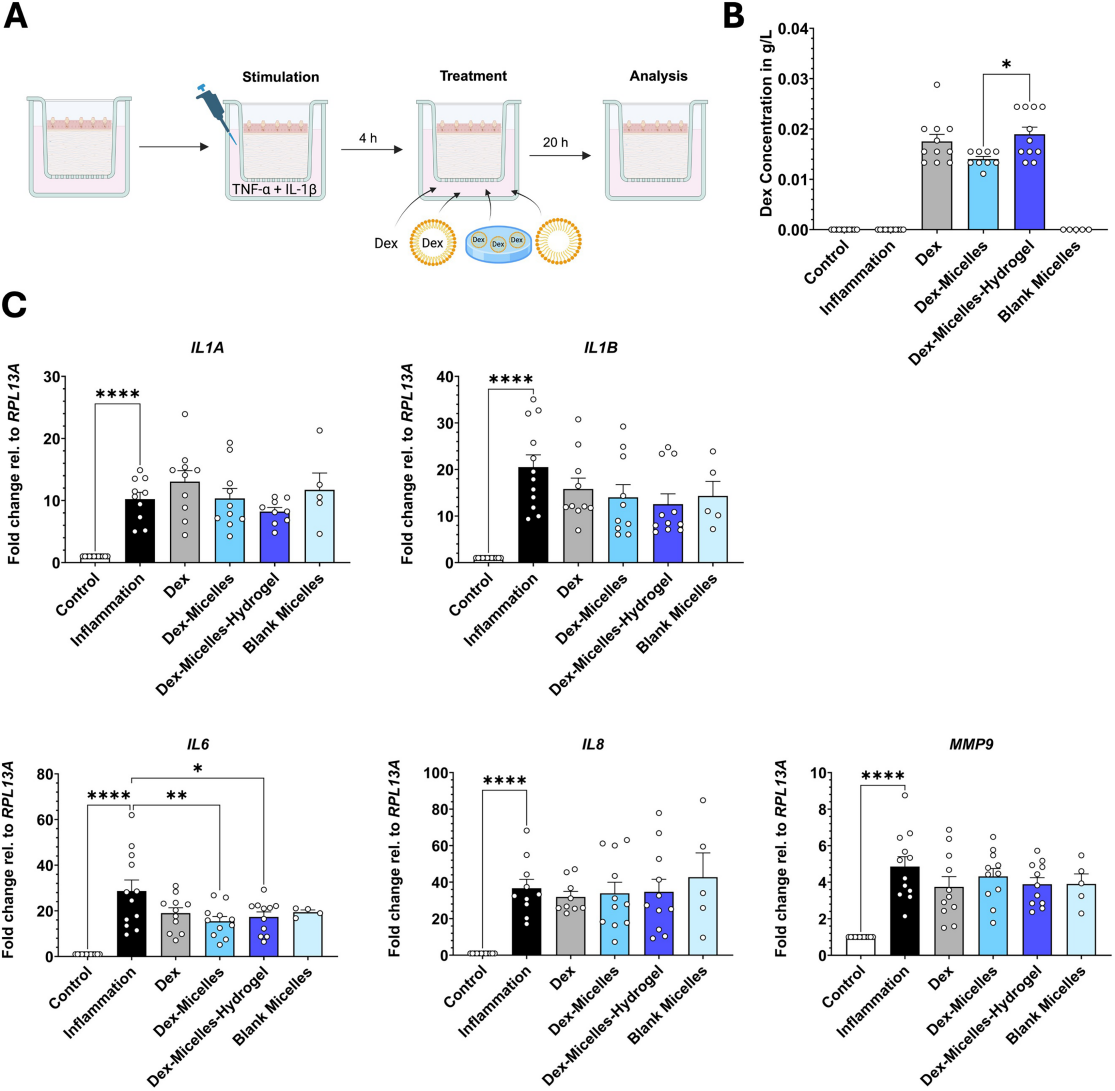
Full-thickness conjunctiva model as test platform for new treatment options. **(A)** Graphical abstract of the application protocol. Created in BioRender. Salman Haider, M. (2025) https://BioRender.com/fttldn3. **(B)** HPLC of Dex in cell culture supernatants. **(C)** RT-qPCR of pro-inflammatory markers after treatment with Dex-formulations. *P < 0.05, **P < 0.01, ***P < 0.001, ****P < 0.0001.

After successful establishment of a FTConM with a DED-like inflammatory cytokine expression profile by the combinatorial administration of TNF-α and IL-1β, we used this model to test the anti-inflammatory effect of a novel hydrogel-based DDS (Fig. 4A). The vehicle system consisted of a modified hyaluronic hydrogel in combination with POx-based Dex-loaded micelles, which allows a more controllable drug release. Pristine Dex, Dex-loaded micelles, and blank micelles were used as controls. Initially, Dex release in cell culture models was quantified using HPLC. The measured concentrations were comparable across the experimental groups (Fig. 4B). However, Dex levels were notably lower in the Dex-Micelles group compared to pristine Dex and the Dex-micelles-hydrogel group. This reduction may be due to increased cellular uptake of micelles or enhanced retention of the micelles within the collagen-based stroma.

Following this, we investigated the anti-inflammatory effect of the Dex-micelles-hydrogel by analyzing cytokine gene expression of inflamed FTConM. In line with the previous experiments, stimulation with TNF-α + IL-1β led to an increased cytokine expression profile (Fig. 4C). Treatment with the Dex-micelles-hydrogel resulted in a decrease of *IL6* expression compared to the inflamed, non-treated condition. Dex-Micelles exhibited a similar effect as the Dex-micelles-hydrogel, including a significantly reduced reduction in *IL6* expression.

**Figure 5.**
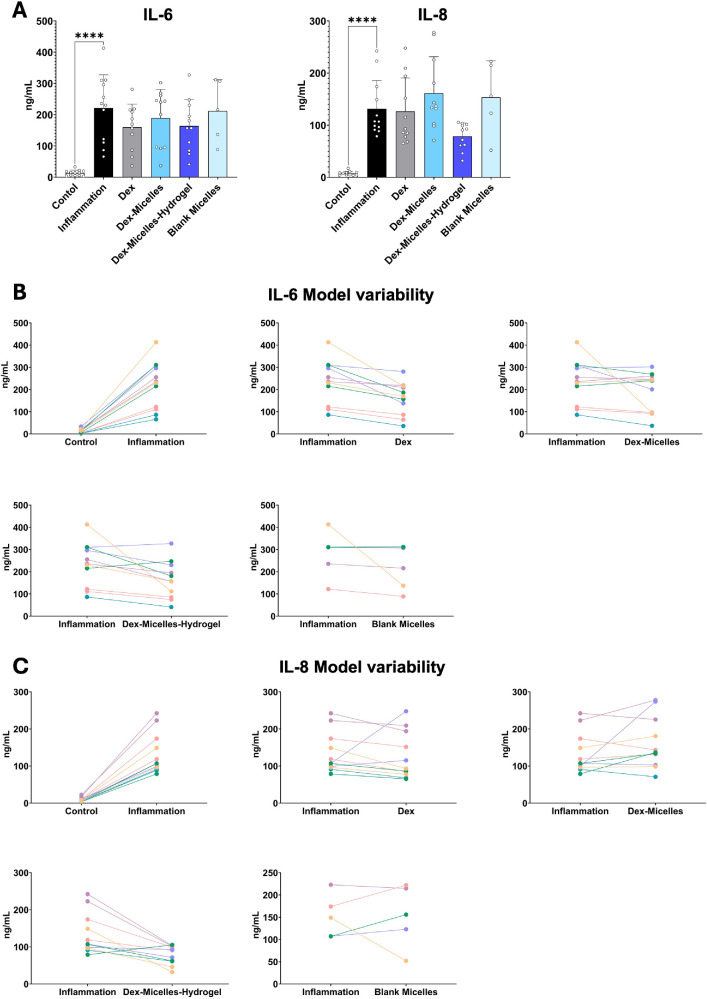
Analysis of model variability via ELISA of IL-6 and IL-8. **(A)** ELISA of IL-6 and IL-8 of all measured data points. **(B)** Single experiment analysis of different models of varying donors for IL-6. **(C)** Single experiment analysis of different models of varying donors for IL-8. *P < 0.05, **P < 0.01, ***P < 0.001, ****P < 0.0001.

To gain a more comprehensive picture of the effect of the Dex-micelles-hydrogel on the initial cellular inflammatory response, we investigated the secretion of IL-6 and IL-8 using ELISA (Fig. 5A). We observed high standard deviations between models of different donors. Overall, we measured a range of IL-6 secretion from 66 ng/mL to 413 ng/mL and a range of IL-8 secretion from 79 ng/mL to 242 ng/mL after cytokine stimulation. After treating the models with the Dex-micelles-hydrogel, reductions reached from 17% to 73% for IL-6 and from 6% to 79% for IL-8 in single experiment analyses, despite statistical insignificance. As in research and development of anti-inflammatory drugs, variations in drug responsiveness by patients are documented,^36^^,^^37^ we analyzed how individual models reacted to the stimulation and treatments. (Figs. 5B, 5C). The analysis showed that most models that show a high responsiveness in terms of an IL-6 an IL-8 increase also effectively responded to the Dex-micelles-hydrogel treatment with a decrease in cytokine release. Similarly, models that showed a smaller increase of IL-6 and IL-8 also reacted to the treatment to a lesser extent. On the other hand, some models did not react to the treatment, which indicates varying treatment responsiveness or possible varying response times of primary cells.

**Figure 6.**
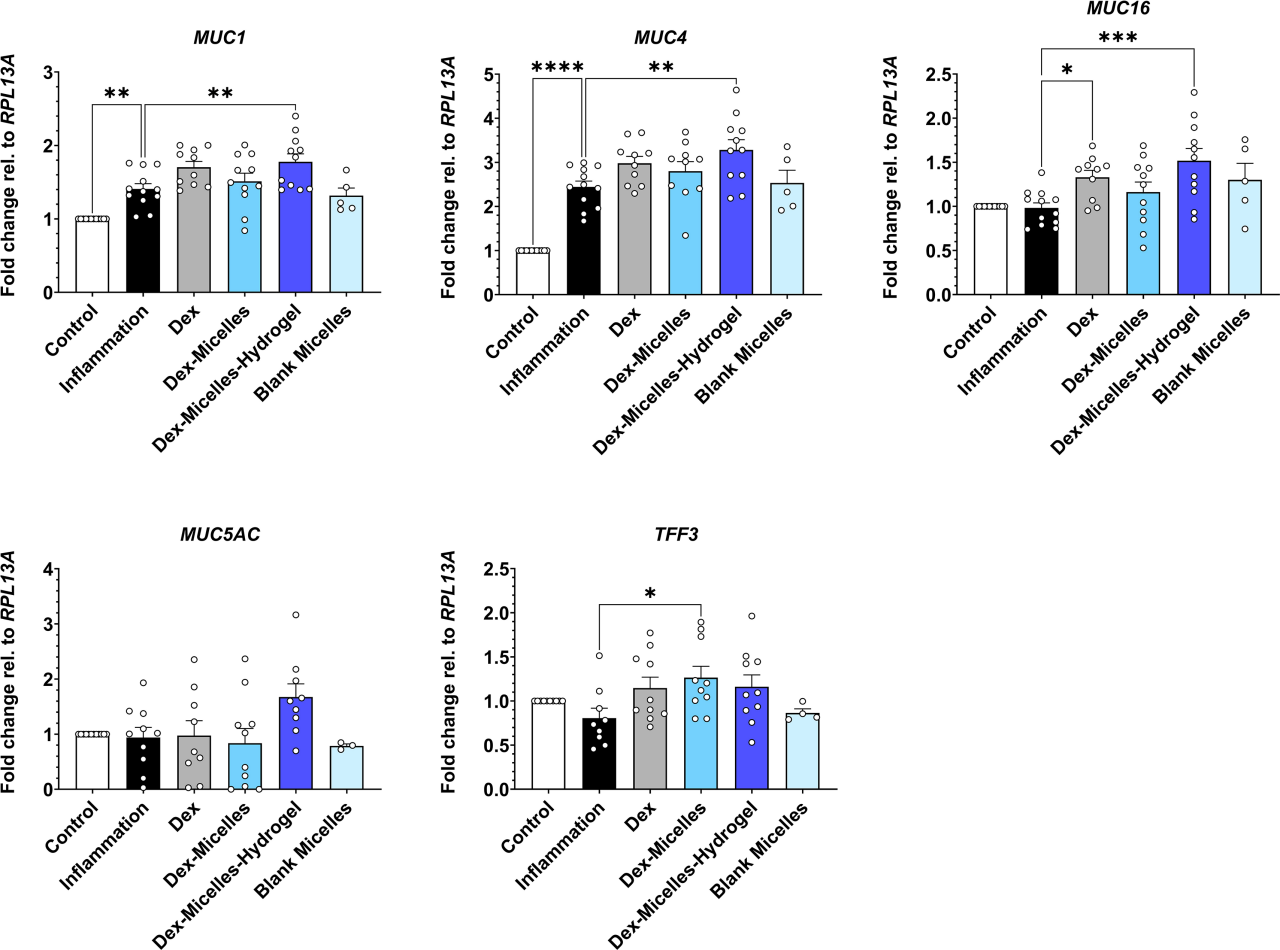
RT-qPCR analysis of conjunctival mucins and trefoil factor 3 (TFF3) after inflammation and treatment with Dex-formulations. *P < 0.05, **P < 0.01, ***P < 0.001, ****P < 0.0001.

Next to an increased cytokine release, another important factor of DED pathophysiology is the expression of mucins which contribute to tear film stability.^8^^–^^10^ Investigating mucin gene expression, we observed a significant increase in the expression of the MAMs *MUC1* and *MUC4*, but not for *MUC16* in TNF-α + IL-1β–stimulated models compared to the unstimulated control (Fig. 6). The Dex-micelles-hydrogel significantly increased the expression of *MUC1*, *MUC4*, and *MUC16* compared to the inflamed, non-treated control while this increasing effect was not significant for the expression of *MUC5AC* (*P* = 0.06)*.* The application of other tested Dex formulations resulted in a significant increase of *MUC16* expression on treatment with Pristine Dex. We additionally analyzed the expression of *TFF3,* which is secreted by goblet cells and further stabilizes the tear film.^38^ Interestingly, only treatment with Dex-micelles resulted in a significant increase in *TTF3* expression compared to the inflammation control. Overall, our data show a general increasing effect of Dex on the expression of MAMs and indicate that administration of Dex via the Dex-micelles-hydrogel can reinforce this effect.

## Discussion

In this study, we showed that our 3D conjunctival model reacts to inflammatory stimuli, as well as to the subsequent treatment with Dex formulations. In addition to the analysis of pro-inflammatory marker expression, we showed that conjunctival mucins can be assessed in our model under inflammatory conditions, which opens an interesting tool to investigate DED related changes in mucin expression. As TNF-α and IL-1β are commonly applied as single cytokines in various inflammation studies,^29^^,^^30^^,^^39^ we investigated them separately and in combination. We found a synergistic effect of the two cytokines on DED-associated pro-inflammatory marker expression.^40^^,^^41^ This could be taken into consideration in DED in vitro modeling, as DED can have different severities and subtypes in patients with varying levels of inflammatory markers.^40^^,^^41^ Additionally, analysis on Ki67 staining showed that the application of cytokines significantly reduced Ki67 positive epithelial cells in our model, whereas viability was not reduced as seen in the MTT assay. Although some studies report increased Ki67 under DED conditions,^42^^,^^43^ its decrease in our model could correlate with a switch to a less proliferative, more differentiated phenotype, because Ki67 typically increases when differentiation decreases.^44^ This correlates with the measured increase of MAM expression on inflammation in our model, indicating enhanced differentiation and reduced proliferative activity.

Inflammation of the ocular surface is commonly treated by the topical application of eye drops with corticosteroids like Dex, prednisolone, fluormetholone, or loteprednoletabonate.^45^^–^^47^ However, eye drops are limited in their efficacy due to a low bioavailability and low patient compliance.^13^^–^^15^ The amphiphilic structure of micelles has been shown to increase bioavailability and solubility while hydrogels have been gaining immense interest in the past years as DDS.^48^^,^^49^ In our study, we used our conjunctival model to test a novel hydrogel DDS that was loaded with Dex-micelles. By treating inflamed models with the hydrogel, a significant reduction of *IL6* expression was measured. Although the hydrogel had a detectable anti-inflammatory effect, we observed high variance throughout experiments. These effects possibly derive from the use of primary cells from varying donors. Overall, we observed high-responding and low-responding models to both the inflammatory stimulus and to the applied treatment. Because patients show varying responsiveness to drugs,^36^^,^^37^ it would be interesting to document possible correlations of high- and low-responding primary cells to the responsiveness of their donor. Although in this study donor samples were entirely anonymized, it could be beneficial in the future to include information about sex and age of donor material, due to the varying sensitivity and responsiveness to drugs between men and women as well as between age groups.^50^^–^^53^ A further aspect that could be taken into consideration is the donor's exposure to cigarette smoke and air pollution, because growing evidence suggests that these factors induce resistance to glucocorticoids.^54^ In future it could also be tested whether low-responding models react more to the treatment when exposed to it for a longer period of time or if weaker cytokine stimulation and the use of non-steroidal anti-inflammatory drugs cause different outcomes. Regarding the specific time-related expression of pro-inflammatory cytokines that is described in literature, further time points of our tested treatments will be analyzed in the future as well.^55^^,^^56^ Because changes in mucin expression are involved in the pathophysiology of DED,^57^ analyses on MAMs and secreted mucins are important upon the induction of inflammation and the treatment with anti-inflammatory drugs, especially due to the distinct observations of mucin levels in DED. Regarding MAMs, decreased levels of *MUC1*, *MUC4*, and *MUC16* have been reported in one group of DED patients^8^^–^^10^ whereas in others, soluble MUC16 and as soluble and membrane-bound MUC1 was increased.^58^^,^^59^ Generally, it has been demonstrated that TNF-α induces the expression of *MUC1* and *MUC4*, which was also the case in our model, suggesting that their reduction in DED originates from other mechanisms.^12^ The treatment with the Dex-micelles-hydrogel significantly increased the expression of all three MAMs compared to untreated inflamed models. This could be an interesting medication approach for DED patients who show a decrease of MAMs.

Although our model was able to react to inflammatory cytokines and Dex formulations, the addition of immune cells is important to recreate a more holistic immune response and increase model predictivity. In DED patients, an increased proportion of neutrophils, leukocytes, CD4 and CD8 T cells are reported,^60^ whereas higher concentrations of T helper cell (Th) 1 and Th17 derived pro-inflammatory cytokines further promote inflammation.^61^^–^^64^ Dex has been shown to promote a shift from Th1 into Th2 cytokines, thereby reducing the pro-inflammatory response of immune cells.^65^^,^^66^ Because of Dex's inhibitory effect on the NF-κB pathway,^67^^,^^68^ it can still be used in in vitro models without the presence of immune cells; however, as a next step in model development, the incorporation of peripheral blood mononuclear cells in inflammatory studies would be of interest.

Overall, we showed that initial reactions to inflammatory stimuli can be assessed in our 3D conjunctival model. For the investigation of poorly understood mechanisms of mucin regulation in DED, the possibility to analyze mucin expression under pro-inflammatory conditions in a complex 3D model enables a deeper investigation of the interplay between DED and conjunctival mucin expression in the future.
